# Reported Characteristics of Anaphylaxis Associated With Glatiramer Acetate: A Pharmacovigilance Analysis of the FDA and Canadian Databases

**DOI:** 10.1002/prp2.70303

**Published:** 2026-07-31

**Authors:** Tailei Nie, Hongyu Liu, Chuanxu Cheng, Jian Li, Jiajing Lu

**Affiliations:** ^1^ Department of Pharmacy, Affiliated Hospital of Yangzhou University Yangzhou University Yangzhou Jiangsu China; ^2^ Department of Biomedical Engineering Columbia University New York New York USA; ^3^ School of Biomedical Sciences and Engineering South China University of Technology Guangzhou Guangdong China; ^4^ Department of Pharmaceutics, School of Pharmacy China Pharmaceutical University Nanjing China

**Keywords:** anaphylaxis, CVAR, FAERS, glatiramer acetate, pharmacovigilance

## Abstract

The FDA has mandated a Boxed Warning for glatiramer acetate (GA) due to the risk of serious anaphylactic reaction. This study aimed to characterize the reported features of GA‐associated anaphylaxis. Data mining was performed using the Reporting Odds Ratio (ROR) in the FDA Adverse Event Reporting System (FAERS) database (Q1 2004–Q1 2025) and the Canadian Vigilance Adverse Reaction (CVAR) database (Q1 1996–Q1 2025). Demographic characteristics, time‐to‐onset (TTO), and case seriousness were compared using the Mann–Whitney *U* test or the Chi‐squared (*χ* ^2^) test. In the FAERS database, 9816 reports were identified with 29 significant disproportionality signals. Younger age (*p* < 0.01) was significantly associated with a higher proportion of serious reports. The association between GA and anaphylactic AEs remained statistically significant after stratification by age, body weight, sex, and reporter types. Twenty AEs demonstrated significantly higher reporting rates in serious cases (*p* < 0.05). Clinical prioritization identified 9 moderate‐priority and 20 weak‐priority signals. The median TTO was 197 days for moderate‐priority signals versus 153 days for weak‐priority signals. All signals exhibited early failure‐type patterns, indicating that reported anaphylactic events were more frequently observed earlier after treatment initiation than later in treatment. In the CVAR database, 711 reports were identified. A total of 17 PTs overlapped with FAERS, with respiratory arrest uniquely present in CVAR. Three PTs were rated as moderate priority in the CVAR database but weak priority in FAERS. This large‐scale pharmacovigilance analysis of the FDA and Canadian databases characterizes the safety signals derived from GA‐associated reports of anaphylaxis, providing a hypothesis‐generating foundation for future pharmacovigilance validation.

## 1 Introduction

GA, a synthetic polypeptide that mimics myelin basic protein, has been a first‐line disease‐modifying therapy for relapsing–remitting multiple sclerosis (MS) since its approval by the FDA in 1996 [1, 2]. Its mechanism of action, which involves inducing regulatory T‐cells to suppress neuroinflammation, offers favorable efficacy with historically fewer systemic risks than interferon‐based therapies or newer immunosuppressants [3]. However, post‐marketing surveillance has identified rare but potentially severe hypersensitivity reactions, leading the FDA to issue a 2025 Boxed Warning regarding the risk of anaphylaxis [4]. This regulatory step underscores significant gaps in understanding the real‐world safety profile of this drug.

Anaphylaxis is a medical emergency characterized by rapid onset and multisystem hypersensitivity, affecting the respiratory system (bronchospasm, laryngeal edema), cardiovascular stability (hypotension, tachycardia), and skin (urticaria, angioedema) [5]. Mortality rates approach 1%, despite the availability of epinephrine, with delayed recognition being a major factor [6]. GA's molecular structure—consisting of glutamic acid, lysine, alanine, and tyrosine—may function as a hapten, binding to endogenous proteins to form neoantigens that induce IgE‐mediated mast cell degranulation [7]. Cross‐reactivity between GA epitopes and myelin autoantigens could further enhance immune activation in susceptible MS patients [8]. Paradoxically, although intended to modulate autoimmunity, this immunogenicity may also contribute to its potential for causing anaphylaxis.

Current evidence on GA‐associated anaphylaxis remains alarmingly limited. Clinical trials, powered for efficacy rather than rare adverse AEs, reported hypersensitivity incidence at 0.5%–1.5%, but methodological constraints obscure real‐world epidemiology [9, 10]. Existing pharmacovigilance studies suffer from:

1. Temporal limitations: Inadequate follow‐up duration to capture delayed reactions;
2. Case misclassification: Broad MedDRA terms (e.g., “hypersensitivity”) diluting anaphylaxis‐specific signals;
3. Unstratified analyses: Failure to account for age, sex, or comorbidity effects on severity;
4. Inadequate clinical contextualization: Isolated disproportionality metrics without prioritization of life‐threatening events.

Consequently, clinicians lack evidence‐based guidance on risk stratification, monitoring protocols, or intervention timelines.

Pharmacovigilance databases like the FAERS and CVAR databases offer unprecedented power to detect rare AEs through disproportionality analysis [11]. The reporting odds ratio (ROR) quantifies signal strength by comparing target drug‐event reporting frequency against background incidence [12]. When coupled with temporal analytics (time‐to‐onset, Weibull distribution modeling) and multidimensional clinical prioritization (fatality rates, designated medical events), this approach transforms raw AE data into actionable risk intelligence. Yet no comprehensive study has applied this integrated framework to GA‐associated anaphylaxis—a critical gap given its Boxed Warning status.

This study leverages data from the FAERS and CVAR databases to address four pivotal aims:

1. Signal Detection: Identify and validate anaphylactic signals through ROR and 95% confidence intervals across 87 Preferred Terms (MedDRA v22.1), with stratification by age, sex, weight, and reporter type;
2. Clinical Phenotyping: Characterize demographic predictors, seriousness drivers, and outcomes of GA‐associated reports;
3. Signal Prioritization: Implement a semiquantitative scoring matrix evaluating reporting rates, signal stability, fatality, and clinical relevance (DME/IME status) to classify 29 signals into moderate/weak priority tiers;
4. Temporal Analytics: Characterize time‐to‐onset (TTO) patterns using Weibull shape parameters to identify temporal windows with higher reporting frequencies.

By integrating disproportionality analytics, clinical prioritization, and temporal dynamics, this analysis provides the characterization of GA‐associated anaphylaxis reporting patterns, offering hypothesis‐generating insights that warrant further investigation to inform post‐marketing safety surveillance and future safety evaluation strategies.

## 2 Materials and Methods

### 2.1 Data Source and Collection

**FIGURE 1:**
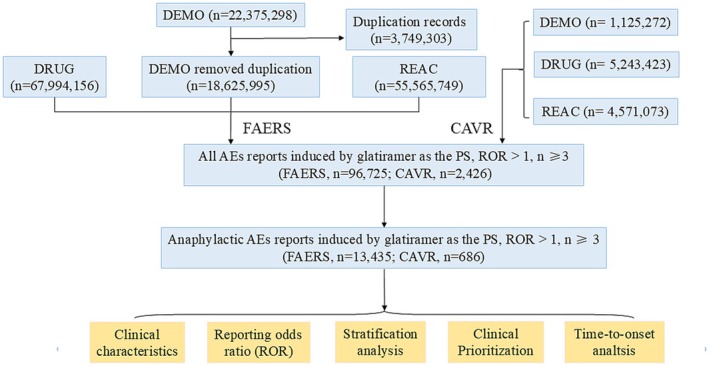
The process of selecting GA associated anaphylactic AEs from the FAERS and CAVR databases. *n*, number of cases with suspected AEs associated with the target drug; ROR, reporting odds ratio.

The pharmacovigilance study of GA‐associated anaphylaxis using post‐marketing data from the FAERS database (Q1 2004–Q1 2025) and CVAR database (1996–2025). FAERS adheres to International Council for Harmonization (ICH) E2B safety reporting guidelines and codes adverse events using the Medical Dictionary for Regulatory Activities (MedDRA) terminology. Duplicate reports were resolved by [13] (1) retaining the latest submission (FDA_DT) for identical CASEID entries; (2) selecting the highest PRIMARYID when CASEID and FDA_DT match. AEs attributed to GA were identified at both System Organ Class (SOC) and Preferred Term (PT) levels. Drugs were categorized by reported role: primary suspect (PS), secondary suspect (SS), concomitant (C), or interacting (I). Target drugs included GA generics (*glatiramer*) and trade names (*Copaxone, Glatopa*); only AEs where GA was designated as PS were analyzed. This study removed duplicate cases and conducted disproportionality analysis in accordance with FAERS methods. A flow diagram including the multi‐step process of data extraction, processing, and analysis was shown in Figure 1.

### 2.2 Statistical Analysis

The ROR, a key algorithm in disproportionality analysis, was calculated based on a 2 × 2 contingency table (Table S1). We included only anaphylactic AEs with at least three reported cases and ROR > 1. Signal strength for GA‐associated reports of anaphylaxis in the FAERS database was assessed, with a positive signal defined as a lower limit of the ROR 95% confidence interval (CI) greater than one [14].

Further compared serious and non‐serious reports to evaluate the severity of the detected safety signals and identify potential risk factors, including gender, age, and weight. Categorical variables were analyzed using Pearson's chi‐square (*χ* ^2^) or Fisher's exact test, while continuous non‐normally distributed data (e.g., age, weight) were assessed using the Mann–Whitney *U* test. All analyses were conducted in R 4.4, with statistical significance set at *p* < 0.05.

To investigate whether stratification factors influenced the association between GA and anaphylactic AEs, we performed subgroup analyses by gender (female, male), age (18–64, > 65 years), weight (< 80, 80–100, > 100 kg), and reporter type (healthcare professional, consumer).

### 2.3 Signal Prioritization Methodology

AEs emerging with a significant association in at least one disproportionality analysis were ranked based on a semiquantitative score assessing the criteria listed in Table S2. The criteria contain [15]: clinical relevance, reporting frequency, signal stability, and case fatality rate.

### 2.4 Time‐To‐Onset Analysis

Time‐to‐onset (TTO) was defined as the interval between the AEs onset date (EVENT_DT in DEMO file) and start date of GA use (START_DT in THER file) [16]. To ensure the accuracy of this calculation, reports with input errors (EVENT_DT earlier than START_DT), inaccurate date entries, and missing specific data were excluded. The medians, quartiles, and the Weibull shape parameter (WSP) test were used to evaluate the TTO in our study [17]. The TTO statistical analysis was conducted using the WSP test, which could determine the varying ratio of incidence of AEs. The shape of the Weibull distribution was described by two parameters: scale (α) and shape (β). To evaluate whether the reporting frequency of these AEs over time, we calculated the median TTO and WSP of signals with strong, moderate, or weak clinical priority after GA use. The selection of parameters and criteria for evaluation was described in previous studies. All WSP tests were performed by R 4.4.

### 2.5 Adverse Event and Drug Identification

According to Medical Dictionary for Regulatory Activities (MedDRA, version 22.1) at the Preferred Term level, anaphylactic symptoms were chosen from the REAC files. All the anaphylactic PTs were listed in Table S3.

## 3 Results

### 3.1 Descriptive Analysis

During the study period, a total of 18 625 995 AE reports were retrieved from the FAERS database after deduplication, including 13 435 reports of GA‐associated anaphylaxis affecting 9816 patients. Females predominated (7945, 88.3%) over males (1498, 11.7%). Anaphylactic reports occurred primarily in middle‐aged patients (18–65 years, *n* = 5251, 91.2%). The proportions of body weight > 100, 50–100, and < 50 kg were 16.6%, 79.3%, and 4.1%. Serious outcomes of anaphylactic and overall reports were recorded in 4052 and 20 482. Additionally, 63.2% of anaphylaxis reports were submitted by healthcare professionals (*n* = 5349, 63.2%), compared with 36.8% submitted by consumers (*n* = 3119, 36.8%). Multiple sclerosis (*n* = 6375, 79.8%) was the most reported indication. The country with the most anaphylactic reports (*n* = 7555, 77.4%) was the USA.

The characteristics of the two database cases are largely comparable. In the CVAR database, 1 125 272 records were analyzed, identifying 711 reports of GA‐associated anaphylaxis. Females comprised the majority of cases (302, 83.4%). Anaphylactic AEs occurred most frequently in the middle‐aged group (304, 95.6%). In terms of body weight, 86.4% of patients weighed between 50 and 100 kg, while 13.7% weighed over 100 kg. A total of 57 serious anaphylactic reactions and 191 serious overall AEs were reported. Healthcare professionals and consumers submitted a comparable number of anaphylaxis reports. The detailed characteristics were summarized in Table 1.

**TABLE 1:** Reported characteristics of patients with GA‐associated anaphylactic AEs from FAERS and CVAR databases.

Characteristics	FAERS		CVAR	
	Anaphylactic AEs*n* = 9816	Overall AEs*n* = 51 433	Anaphylactic AEs*n* = 365	Overall AEs*n* = 986
	Available number	Available number	Available number	Available number
*Gender, n (%)*				
Female	7945 (88.3%)	39 272 (80.8%)	302 (83.4%)	782 (80.5%)
Male	1498 (11.7%)	9331 (19.2%)	60 (16.6%)	189 (19.5%)
*Age (years), n (%)*				
< 18	75 (1.3%)	325 (1.3%)	3 (0.9%)	10 (1.2%)
≤ 18 and ≤ 65	5251 (91.2%)	21 125 (87.3%)	304 (95.6%)	760 (94.8%)
> 65	432 (7.5%)	2738 (11.3%)	11 (3.5%)	32 (4.0%)
*Weight (kg), n (%)*				
< 50	57 (4.1%)	247 (5.3%)	0	8 (10.7%)
≤ 50 and ≤ 100	1116 (79.3%)	3716 (79.5%)	19 (86.4%)	62 (82.7%)
> 100	234 (16.6%)	713 (15.2%)	3 (13.7%)	5 (6.6%)
*Reported countries, n (%)*				
United States	7555 (77.4%)	41 576 (81.2%)	—	—
Non‐United States	2208 (22.6%)	9628 (18.8%)	—	—
*Indications, n (%)*				
Multiple Sclerosis	6375 (79.8%)	31 953 (62.1%)	—	—
Relapsing–remitting Multiple sclerosis	597 (6.1%)	2038 (4.0%)	—	—
Others	1021 (12.8%)	9295 (21.5%)	—	—
*Outcomes, n (%)*				
Non‐serious outcome	5764 (58.7%)	30 951 (60.2%)	162 (74.0%)	335 (63.7%)
Serious outcome	4052 (41.3%)	20 482 (39.8%)	57 (26.0%)	191 (36.3%)
*Reporters, n (%)*				
Health professional	5349 (63.2%)	16 955 (38.0%)	67 (37.4%)	257 (47.5%)
Consumer	3119 (36.8%)	27 705 (62.0%)	112 (63.6%)	284 (52.5%)
*Reporting year, n (%)*				
Before 2004			62 (17.0%)	135 (14.0%)
2004–2010	598 (6.1%)	2618 (5.1%)	43 (11.8%)	120 (12.5%)
2011–2015	2369 (34.1%)	12 048 (23.4%)	66 (18.1%)	236 (24.5%)
2016–2020	5143 (52.4%)	27 496 (53.4%)	112 (30.7%)	286 (29.7%)
2021–2015	1706 (17.4%)	9235 (18.0%)	82 (22.4%)	185 (19.2%)

Abbreviations: AEs, adverse events; *n*, number of cases.

### 3.2 Disproportionality Analysis

**FIGURE 2:**
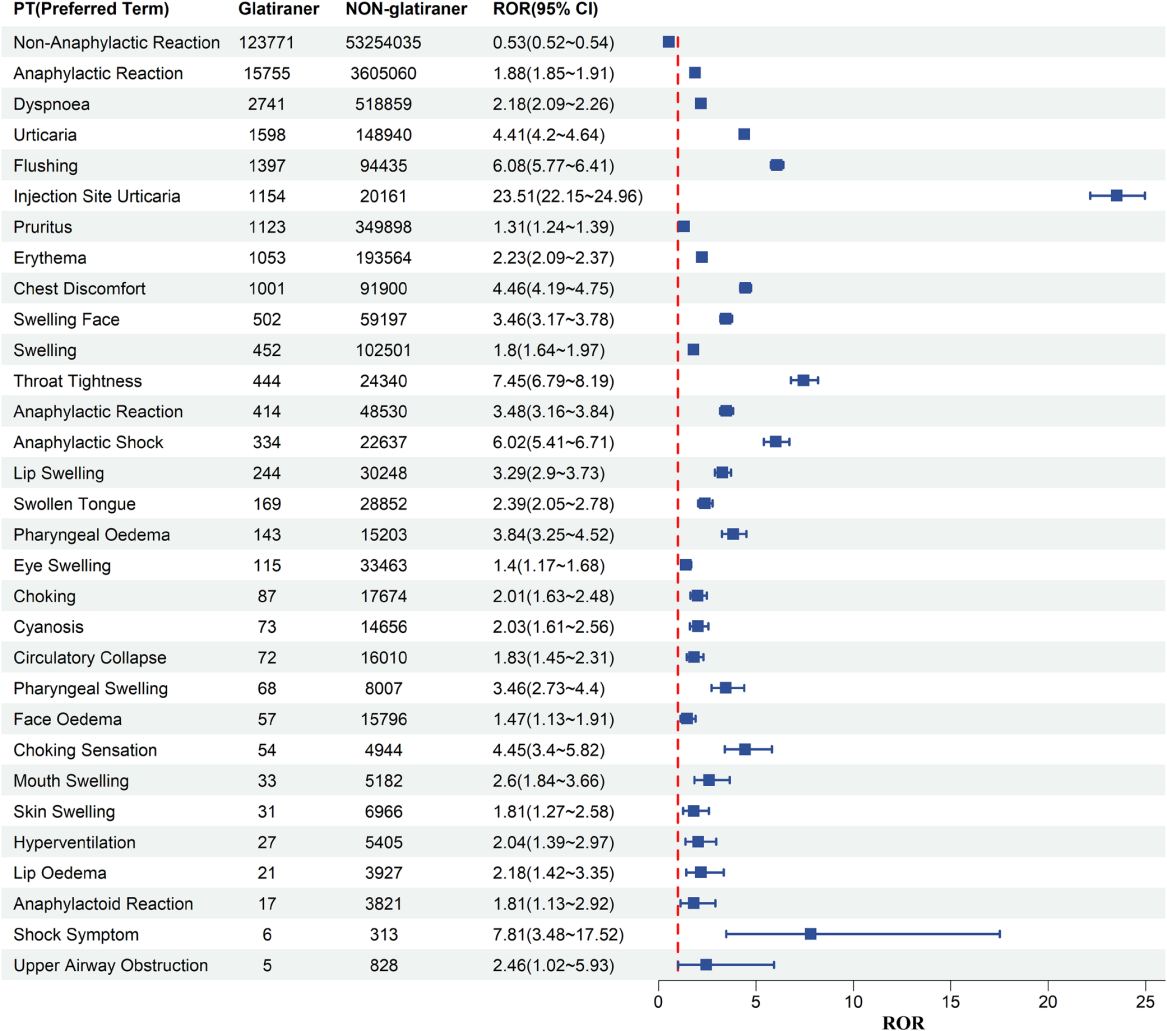
Reporting odds ratios (ROR) with 95% CI for all positive GA related anaphylactic AEs in the FAERS database. CI, confidence interval; ROR, reporting odds ratio.

A total of 29 different PTs of the anaphylactic reports associated with GA were reported in the FAERS database (Figure 2), with values of signals ranging from a ROR of 1.31 (95% CI: 1.24–1.39, pruritus) to 23.51 (95% CI: 22.15–24.96, injection site urticaria). The most frequent anaphylactic AEs were dyspnoea (*n* = 2741, ROR = 2.18), urticaria (*n* = 1598, ROR = 4.41), flushing (*n* = 1397, ROR = 6.08). Results of the disproportionality analysis of the anaphylactic reports associated with GA were presented in Figure 2. The report frequency of anaphylactic reaction (*n* = 15 755, ROR = 1.88) related to GA was significantly higher than that of non‐anaphylactic reaction (*n* = 123 771, ROR = 0.53, 95% CI: 0.52–0.54) in the overall database. Among 27 SOCs, the results of Tables S4 and S6 demonstrated that anaphylactic reaction treated with GA exhibited stronger activity than most of the SOCs in these two database.

**FIGURE 3:**
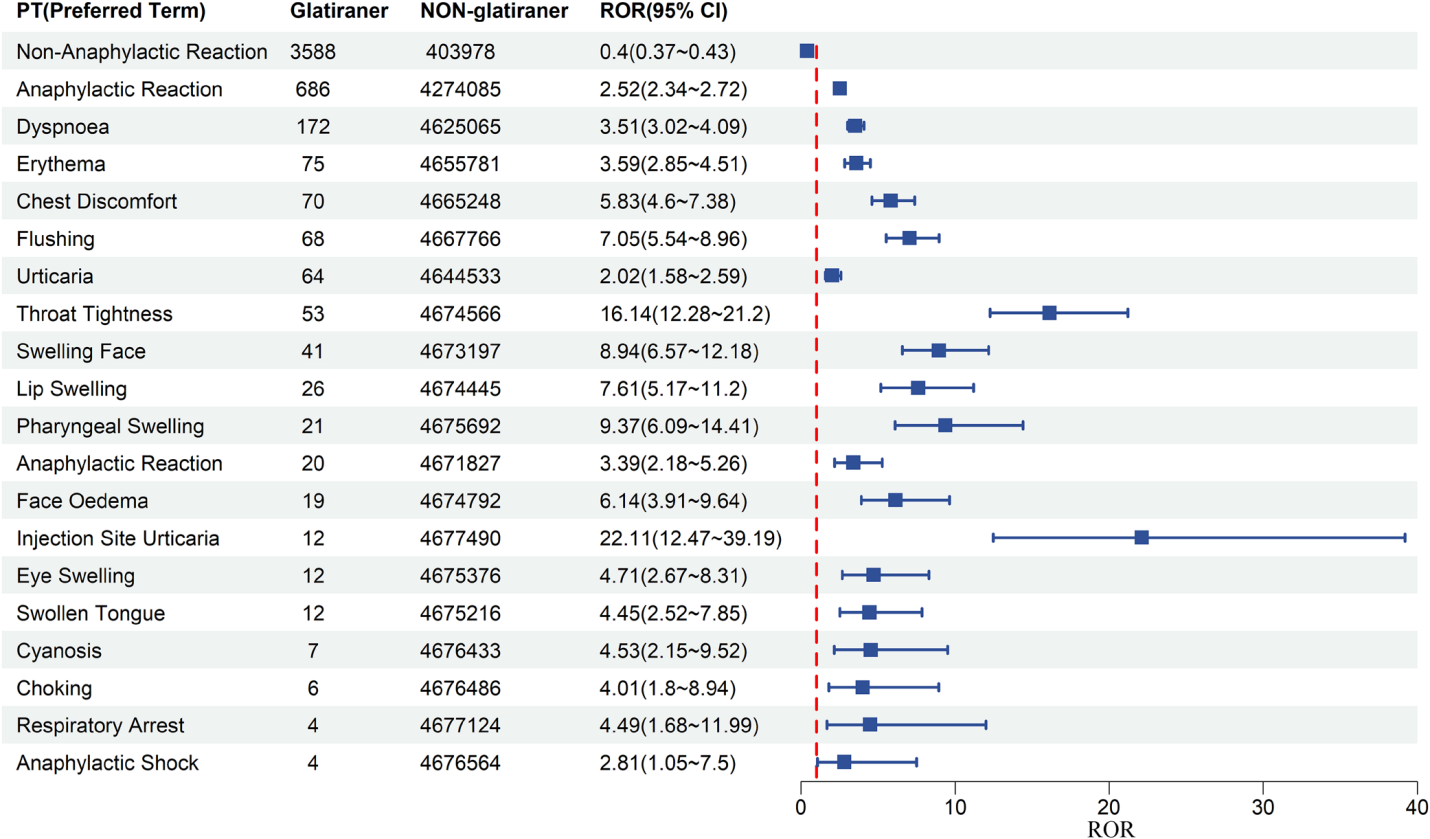
Reporting odds ratios (ROR) with 95% CI for all positive GA related anaphylactic AEs in the CAVR database. CI, confidence interval; ROR, reporting odds ratio.

In the CAVR database, as shown in Figure 3, several adverse reactions exhibit strong safety signals, particularly those related to immediate‐type hypersensitivity. Notably, injection site urticaria shows the highest ROR (*n* = 12, ROR = 22.11), followed by throat tightness (*n* = 53, ROR = 16.14) and pharyngeal swelling (*n* = 21, ROR = 9.37). In contrast, non‐anaphylactic reaction (*n* = 3588, ROR = 0.40) shows a lower incidence relative to anaphylactic reaction (*n* = 855, ROR = 2.52).

A total of 17 PTs were common to both datasets. The CAVR database includes a more restricted set of PTs, with the unique addition of respiratory arrest, which is absent from the FAERS database. Despite these variations, the overlapping terms reinforce the core safety signal of hypersensitivity reactions associated with GA.

### 3.3 Serious Versus Non‐Serious Cases

Significant differences emerged between serious and non‐serious anaphylactic reaction cases in patients receiving GA (Table 2). Serious cases occurred in younger patients (median age 48 years, IQR 37–58 vs. 51 years, IQR 39–58; *p* < 0.01), while sex (*p* = 0.97) and body weight (*p* = 0.84) showed no significant intergroup differences. Twenty AEs demonstrated significantly higher reporting rates in serious cases (*p* < 0.05), including dyspnoea (*χ* ^2^ = 73.76, *p* < 0.01), chest discomfort (*χ* ^2^ = 22.98, *p* < 0.01), erythema (*χ* ^2^ = 55.97, *p* < 0.05), swelling face (*χ* ^2^ = 88.61, p < 0.01). Conversely, six AEs were predominantly reported in non‐serious cases: flushing (*χ* ^2^ = 53.51, *p* < 0.01), urticaria (*χ* ^2^ = 346.09, p < 0.01). Notably, all reported shock events (*n* = 6) resulted in serious outcomes.

**TABLE 2:** Differences in clinical characteristics of serious and non‐serious reports.

	Serious cases	Non‐serious cases	Statistic	*p*
*Gender*				
Female	3324 (41.8%)	4621 (58.2%)	0^b^	0.97^a^
Male	628 (41.9%)	870 (58.1%)	—	—
Age, years (median, IQR)	48 (37–58)	49 (38–58)	−2.73^d^	< 0.01^c^
Weight, kg (median, IQR)	78.28 (62–90.8)	74.84 (63.4–90.7)	−0.21^d^	0.84^c^
*Types of AEs*				
Dyspnoea	1525 (55.64%)	1216 (44.36%)	73.76^b^	< 0.01^a^
Chest discomfort	556 (55.54%)	445 (44.46%)	22.98^b^	< 0.01^a^
Erythema	546 (51.85%)	507 (48.15%)	5.97^b^	< 0.05^a^
Flushing	542 (38.8%)	855 (61.2%)	53.51^b^	< 0.01^a^
Urticaria	417 (26.1%)	1181 (73.9%)	346.09^b^	< 0.01^a^
Pruritus	389 (34.64%)	734 (65.36%)	88.07^b^	< 0.01^a^
Swelling face	346 (68.92%)	156 (31.08%)	88.61^b^	< 0.01^a^
Anaphylactic reaction	294 (71.01%)	120 (28.99%)	87.95^b^	< 0.01^a^
Throat tightness	282 (63.51%)	162 (36.49%)	42.46^b^	< 0.01^a^
Injection site urticaria	244 (21.14%)	910 (78.86%)	363.10^b^	< 0.01^a^
Anaphylactic shock	241 (72.16%)	93 (27.84%)	77.66^b^	< 0.01^a^
Swelling	177 (39.16%)	275 (60.84%)	14.76^b^	< 0.01^a^
Lip swelling	169 (69.26%)	75 (30.74%)	43.31^b^	< 0.01^a^
Swollen tongue	116 (68.64%)	53 (31.36%)	27.85^b^	< 0.01^a^
Pharyngeal edema	101 (70.63%)	42 (29.37%)	28.26^b^	< 0.01^a^
Choking	84 (96.55%)	3 (3.45%)	80.09^b^	< 0.01^a^
Eye swelling	68 (59.13%)	47 (40.87%)	5.14^b^	< 0.05^a^
Circulatory collapse	64 (88.89%)	8 (11.11%)	46.41^b^	< 0.01^a^
Pharyngeal swelling	53 (77.94%)	15 (22.06%)	23.06^b^	< 0.01^a^
Cyanosis	50 (68.49%)	23 (31.51%)	11.33^b^	< 0.01^a^
Choking sensation	48 (88.89%)	6 (11.11%)	34.37^b^	< 0.01^a^
Face edema	40 (70.18%)	17 (29.82%)	10.23^b^	< 0.01^a^
Mouth swelling	24 (72.73%)	9 (27.27%)	7.03^b^	< 0.01^a^
Hyperventilation	20 (74.07%)	7 (25.93%)	6.27^b^	< 0.05^a^
Lip edema	19 (90.48%)	2 (9.52%)	13.43^b^	< 0.01^a^
Anaphylactoid reaction	10 (58.82%)	7 (41.18%)	0.41^b^	0.52^a^
Skin swelling	8 (25.81%)	23 (74.19%)	5.36^b^	< 0.05^a^
Shock symptom	6 (100%)	0	—	—
Upper airway obstruction	4 (80%)	1 (20%)	—	0.20^e^

^a^ Proportions were compared using the Pearson *χ* ^2^ test.

^b^ The *χ* ^2^ statistic of the Pearson chi‐square test.

^c^ Mann–Whitney *U* test.

^d^ The Z statistic of the Mann–Whitney *U* test.

^e^ Fisher's exact test. *p*‐value < 0.05 was considered statistically significant.

### 3.4 Subgroup Analysis

**FIGURE 4:**
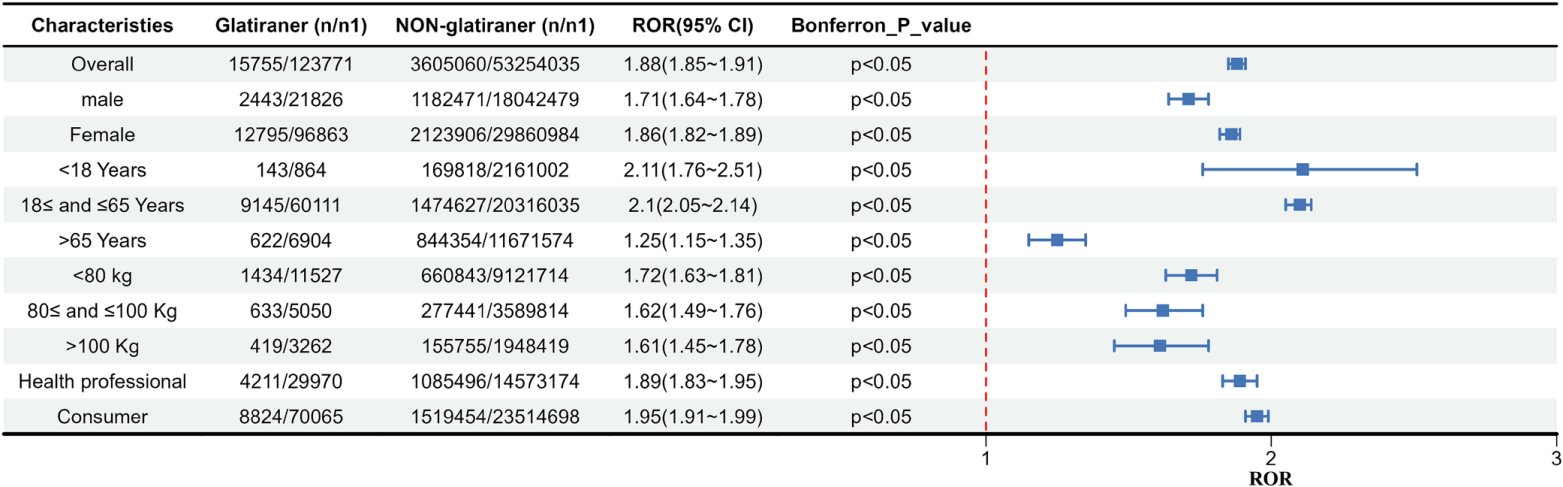
Stratification analysis of GA related anaphylactic AEs in the FAERS database. CI, confidence interval; *N*, number of cases of total AEs associated with the target drug; *n*, number of cases with suspected AEs associated with the target drug; *n*1, number of cases of other drugs; ROR, reporting odds ratio.

We performed subgroup analyses stratified by sex, age, body weight, and reporter type to evaluate the robustness of GA‐anaphylaxis associations. As shown in Figure 4, all stratified subgroups demonstrated consistent disproportionality signals, with RORs remaining statistically significant (lower bounds > 1). These findings confirm a persistent association between glatiramer acetate and anaphylactic reactions across demographic and reporting subgroups.

### 3.5 Clinical Prioritization of Disproportionality Signals

Among 29 significant disproportionality signals (Table 3), 3 (10.3%) represented Important Medical Events (IMEs), while anaphylactic reaction, anaphylactic shock, and anaphylactoid reaction qualified as Designated Medical Events (DMEs). Clinical priority assessment categorized signals as Moderate priority (9 AEs, 31%) and Weak priority (20 AEs, 69%). Anaphylactic reaction and anaphylactic shock were assigned moderate priority, with the highest observed score of 4 (scoring criteria detailed in Table S2). The PTs marked with an asterisk, such as erythema (*n* = 1053) and chest discomfort (*n* = 1001), are rated as moderate priority in the CAVR database but weak priority in the FAERS database, despite having a relatively high number of reports in FAERS. Clinical priority assessment of results for disproportionality signals in the CAVR database is shown in Table S5.

**TABLE 3:** Clinical priority assessing results of disproportionality signals.

PTs	*n*	ROR	Death (*n*)	DMEs/IMEs	Priority level (score)
Injection site urticaria	1154	23.73	0	NA	Weak (2)
Shock symptom	6	8.44	0	IME	Moderate (3)
Throat tightness^a^	444	7.47	0	NA	Weak (2)
Anaphylactic shock	334	6	3	DME	Moderate (4)
Flushing	1397	5.99	0	NA	Moderate (3)
Chest discomfort^**a**^	1001	4.46	2	NA	Weak (2)
Urticaria	1598	4.43	1	NA	Moderate (3)
Choking sensation	54	4.39	0	NA	Weak (2)
Pharyngeal edema	143	3.78	0	NA	Weak (2)
Anaphylactic reaction	414	3.47	1	DME	Moderate (4)
Pharyngeal swelling	68	3.47	0	NA	Weak (2)
Swelling face	502	3.46	0	NA	Weak (2)
Lip swelling	244	3.26	0	NA	Weak (2)
Mouth swelling	33	2.61	0	NA	Weak (2)
Upper airway obstruction	5	2.46	0	IME	Moderate (3)
Swollen tongue	169	2.38	0	NA	Weak (2)
Erythema^a^	1053	2.22	0	NA	Weak (2)
Lip edema	21	2.18	0	NA	Weak (2)
Dyspnoea	2741	2.17	7	NA	Moderate (3)
Choking	87	2.03	1	IME	Moderate (3)
Hyperventilation	27	2.03	0	NA	Weak (2)
Cyanosis	73	1.98	0	NA	Weak (1)
Skin swelling	31	1.87	0	NA	Weak (1)
Anaphylactoid reaction	17	1.82	0	DME	Moderate (3)
Swelling	452	1.8	0	NA	Weak (1)
Circulatory collapse	72	1.77	1	IME	Weak (2)
Face edema	57	1.47	0	NA	Weak (1)
Eye swelling	115	1.4	0	NA	Weak (1)
Pruritus	1123	1.32	0	NA	Weak (1)

*Note:* A score of 0–2, 3–5, and 6–8 was identified, respectively, for AEs with weak, moderate, or strong priority.

Abbreviations: DME, designated medical event; IME, important medical event; PTs, preferred terms; ROR, reporting odds ratio.

^a^ Moderate priority in the CAVR database.

### 3.6 Time‐To‐Onset Analysis

TTO and WSP analyses for the moderate and weak clinical priority signals are shown in Table 4. The median TTO was significantly longer for moderate‐priority signals (197 days, IQR 54–643) than for weak‐priority signals (153 days, IQR 35–592). WPS analysis revealed shape parameters (*β*) < 1 for both signal categories: moderate priority (*β* = 0.63, 95% CI 0.61–0.66), weak priority (β = 0.58, 95% CI 0.56–0.6). Upper 95% confidence limits remained < 1, confirming early failure‐type distributions for both signal categories. This indicates that reported anaphylactic events were more frequently observed earlier after treatment initiation than later in treatment.

**TABLE 4:** Time‐to‐onset analysis for signals with moderate/weak prioritization.

Prioritization				Weibull distribution			
	Case	TTO (days)		Scale parameter		Shape parameter	
	*n*	Median (IQR)	Min‐max	*α*	95% CI	*β*	95% CI
Moderate	1644	197 (54–643)	1–7316	399.53	367.36–431.7	0.63	0.61–0.66
Weak	1824	153 (35–591.25)	1–7502	335.41	307.55–363.27	0.58	0.56–0.6

*Note:* When TTO is 1 day, the adverse event occurred on the same day as the therapy.

Abbreviations: IQR, interquartile range; *n*, number of cases with available time‐to‐onset; TTO, Time‐to‐onset.

## 4 Discussion

This pharmacovigilance study leverages FAERS and CVAR data to characterize GA‐associated anaphylactic reports, addressing critical gaps highlighted by the FDA's 2025 Boxed Warning. Although many studies were consistent with previous clinical trials and literature reviews that GA might increase the risk of anaphylactic reactions, our report presents a more accurate and detailed description and characterization of the anaphylactic AEs spectrum of GA to date, which added stratification analysis, clinical priority of signals, and serious outcomes.

### 4.1 Comparison of Safety Signals Between Different Studies

This study provides a large‐scale pharmacovigilance analysis of GA‐associated reports of anaphylaxis using data from the FAERS and CVAR databases, which complements findings from earlier clinical trials and smaller‐scale studies by real‐world reporting patterns. The current study showed that the most frequently reported anaphylactic AEs were dyspnoea (*n* = 2741, ROR = 2.18), urticaria (*n* = 1598, ROR = 4.41), and flushing (*n* = 1397, ROR = 6.08), which corresponded to clinical trials [18]. The unique PT respiratory arrest identified in the CVAR database constitutes a safety signal suggesting that glatiramer acetate may be associated with a rare but potentially life‐threatening respiratory event. Previous research and clinical trials have reported GA's hypersensitivity incidence at 0.5%–1.5%, with more limited case details provided in safety surveillance data [19, 20].

Our analysis features in‐depth disproportionality analysis and stratification across multiple demographic and reporting variables. The signals identified in this study, including those for dyspnoea (*n* = 2741, ROR = 2.18), urticaria (*n* = 1598, ROR = 4.41), and anaphylactic reactions (*n* = 17, ROR = 1.81), align with previous findings regarding common allergic reactions [21], but the detailed characterization, especially the time‐to‐onset (TTO) analysis, provides new insights. Specifically, the time to onset of anaphylactic reactions ranged significantly between moderate and weak‐priority signals, suggesting that while some reactions occurred earlier (within 153 days), others manifested after a longer delay (up to 197 days). Our research is similar to the FDA's box warning [4]: allergic reactions to this medication may occur up to 6 months after administration. These findings may warrant further investigation regarding the timing of reported allergic reactions and their potential implications for patient monitoring.

This study suggests that while the hypersensitivity risk is well‐recognized, the heterogeneity of reactions and the variability in timing observed in spontaneous reports highlight distinct safety signals. Moreover, unlike the broader category of hypersensitivity in prior studies [22, 23, 24], our research provides a detailed characterization of specific anaphylactic symptoms and their relative reporting associations. These hypothesis‐generating findings warrant further investigation to inform potential patient safety monitoring considerations.

### 4.2 Serious Versus Non‐Serious Reports

A notable finding from our analysis is the difference between serious and non‐serious outcomes among GA‐associated reports of anaphylaxis. Our results showed that while most adverse events were not serious, serious AEs such as dyspnea, anaphylactic shock, and chest discomfort made up a significant part of the reports. Previous studies have rarely addressed these severe AEs [24], and this study fills that gap. Younger age was associated with the reporting of serious versus non‐serious reactions, with serious reports having a lower median age (48 vs. 51 years, *p* < 0.01). Previous studies did not mention differences in the occurrence of adverse reactions based on gender or age [25].

On the other hand, the non‐serious reactions, including urticaria and pruritus, were more frequently reported, which aligns with the general understanding that milder forms of hypersensitivity are more common but less clinically significant [21, 24]. Importantly, our study demonstrated that specific events, such as anaphylactic shock, were strongly associated with serious outcomes, which may warrant further investigation regarding their potential implications for patient monitoring.

These findings suggest that while many reactions are mild and self‐limited, signals of severe hypersensitivity, particularly in younger age groups and during the early phase of treatment, merit further investigation. Given the risk of life‐threatening reactions like anaphylactic shock, future studies should evaluate whether specific symptom patterns are associated with more serious reported outcomes.

### 4.3 Clinical Prioritization of the Disproportionality Signals

The clinical prioritization of safety signals is an essential component of pharmacovigilance, as it allows for the identification of adverse events that require immediate attention. In this study, we classified 29 signals associated with GA‐associated reports of anaphylaxis into moderate and weak clinical priority categories in the FAERS database. Notably, anaphylactic shock and anaphylactic reaction were classified as moderate‐priority signals, given their potential to cause severe, life‐threatening outcomes [26]. Erythema (*n* = 1053) and chest discomfort (*n* = 1001) are rated as moderate priority in the CAVR database but weak priority in the FAERS database, despite having a relatively high number of reports in FAERS. The differential prioritization of PTs, such as throat tightness and chest discomfort, between CAVR and FAERS illustrates the complementary nature of these databases. While FAERS provides a broad‐based safety overview, CAVR contributes regional depth, making it an essential tool for identifying and contextualizing drug safety signals.

This prioritization is consistent with previous studies that emphasize the importance of early detection and rapid intervention in cases of anaphylactic shock [27]. The moderate‐priority classification for signals like dyspnoea and throat tightness underscores the need for clinical vigilance in patients presenting with respiratory distress, as these symptoms can escalate into more severe forms of anaphylaxis [28]. By contrast, the weak‐priority signals, including urticaria and pruritus, were deemed less urgent but still important for monitoring, given their relatively high frequency in the reports [24].

### 4.4 Limitations

Despite the advantages of real‐world data mining strategies utilized in our study based on the FAERS and CVAR databases, there were several limitations inherent in all pharmacovigilance databases. First, only cases with adverse events are included in the databases. The incidence of anaphylactic reports associated with GA cannot be calculated because the total number of patients receiving GA treatment is unknown. Second, the establishment of a definite causal relationship between a target drug and AEs is restricted because disproportionality analysis only provides statistical association. Third, we focus only on AEs in one reaction group, and the deep relationship between GA and other system organ classes remains unknown. Further experimental exploration, clinical trials, case–control studies, and cohort studies are needed to validate the results.

## 5 Conclusion

This pharmacovigilance study presents a comprehensive real‐world analysis of GA‐associated anaphylactic reports, identifying 29 clinically relevant signals, including 9 moderate and 20 weak‐priority events. Key findings reveal that younger age (*p* < 0.01) was associated with a higher proportion of serious anaphylactic AEs, whereas body weight and sex showed no significant correlation with severity. Temporal analysis demonstrated a median TTO of 197 days for moderate‐priority signals and 153 days for weak‐priority signals, with all signals exhibiting early failure‐type patterns, suggesting that reported events occurred more frequently within the first 6 months after treatment initiation. These findings expand the post‐marketing safety knowledge of GA and underscore the need for independent validation through well‐designed pharmacoepidemiologic studies.

## Author Contributions

**Tailei Nie:** writing – original draft, writing – review and editing, methodology. **Jiajing Lu:** conceptualization, investigation, funding acquisition, writing – original draft, writing – review and editing, visualization, validation, methodology, software, formal analysis, project administration, resources, supervision, data curation. **Hongyu Liu:** validation, formal analysis, visualization. **Jian Li:** software, data curation, visualization. **Chuanxu Cheng:** software, data curation, visualization.

## Funding

This work was supported by the Young Scientists Fund of the National Natural Science Foundation of China (Grant No. 82404600).

## Ethics Statement

Since this study used publicly accessible anonymized data from the database, institutional ethical approval was not necessary.

## Conflicts of Interest

The authors declare no conflicts of interest.

## Supporting information


**Table S1:** Calculation of reporting odds ratio (ROR).
**Table S2:** Criteria and relevant scores to prioritize AEs emerged from disproportionality analysis.
**Table S3:** Anaphylactic PTs from MedDRA, version 22.1.
**Table S4:** Signal strength of reports of glatiramer at the System Organ Class (SOC) level in the FAERS database.
**Table S5:** Clinical priority assessment of results for disproportionality signals in the CAVR database.
**Table S6:** Signal strength of reports of glatiramer at the System Organ Class (SOC) level in the CAVR database.

## Data Availability

The original contributions presented in the study are included in the article/Supporting Information, and further inquiries can be directed to the corresponding authors. Publicly available datasets were analyzed in this study. All data from the FAERS database, which is available at https://fis.fda.gov/extensions/FPD‐QDE‐FAERS/FPD‐QDE‐FAERS.html. Data from the CVAR database is available at https://www.canada.ca/en/health‐canada/services/drugs‐health‐products/medeffect‐canada/adverse‐reaction‐database/canada‐vigilance‐online‐database‐data‐extract.html.

## References

[1] The US Food and Drug Administration , “FDA Drug Safety Communication,” (2025), https://www.fda.gov/media/185208/download?attachment.

[2] X. Montalban , R. Gold , A. J. Thompson , et al., “ECTRIMS/EAN Guideline on the Pharmacological Treatment of People With Multiple Sclerosis,” European Journal of Neurology 25, no. 2 (2018): 215–237. 29352526 10.1111/ene.13536

[3] R. Aharoni , “The Mechanism of Action of Glatiramer Acetate in Multiple Sclerosis and Beyond,” Autoimmunity Reviews 12, no. 5 (2013): 543–553. 23051633 10.1016/j.autrev.2012.09.005

[4] W. H. Stuart , S. Cohan , J. R. Richert , and A. Achiron , “Selecting a Disease‐Modifying Agent as Platform Therapy in the Long‐Term Management of Multiple Sclerosis,” Neurology 63, no. 11 Suppl 5 (2004): S19–S27. 10.1212/wnl.63.11_suppl_5.s19 15596732

[5] D. Gonzalez De Olano , W. V. Cain , J. A. Bernstein , et al., “Disease Spectrum of Anaphylaxis Disorders,” Journal of Allergy and Clinical Immunology: In Practice 11, no. 7 (2023): 1989–1996. 37220812 10.1016/j.jaip.2023.05.012

[6] D. de Silva , C. Singh , A. Muraro , et al., “Diagnosing, Managing and Preventing Anaphylaxis: Systematic Review,” Allergy 76, no. 5 (2021): 1493–1506. 32880997 10.1111/all.14580

[7] T. Prod'Homme and S. S. Zamvil , “The Evolving Mechanisms of Action of Glatiramer Acetate,” Cold Spring Harbor Perspectives in Medicine 9, no. 2 (2019): a029249. 29440323 10.1101/cshperspect.a029249 PMC6360864

[8] C. Farina , M. S. Weber , E. Meinl , H. Wekerle , and R. Hohlfeld , “Glatiramer Acetate in Multiple Sclerosis: Update on Potential Mechanisms of Action,” Lancet Neurology 4, no. 9 (2005): 567–575. 16109363 10.1016/S1474-4422(05)70167-8

[9] M. Rastkar , M. Ghajarzadeh , and M. A. Sahraian , “Adverse Side Effects of Glatiramer Acetate and Interferon Beta‐1a in Patients With Multiple Sclerosis: A Systematic Review of Case Reports,” Current Journal of Neurology 22, no. 2 (2023): 115–136. 38011449 10.18502/cjn.v22i2.13340 PMC10460926

[10] H. Rauschka , C. Farina , P. Sator , S. Gudek , F. Breier , and M. Schmidbauer , “Severe Anaphylactic Reaction to Glatiramer Acetate With Specific IgE,” Neurology 64, no. 8 (2005): 1481–1482. 15851756 10.1212/01.WNL.0000158675.01711.58

[11] T. Sakaeda , A. Tamon , K. Kadoyama , and Y. Okuno , “Data Mining of the Public Version of the FDA Adverse Event Reporting System,” International Journal of Medical Sciences 10, no. 7 (2013): 796–803. 23794943 10.7150/ijms.6048 PMC3689877

[12] K. J. Rothman , S. Lanes , and S. T. Sacks , “The Reporting Odds Ratio and Its Advantages Over the Proportional Reporting Ratio,” Pharmacoepidemiology and Drug Safety 13, no. 8 (2004): 519–523. 15317031 10.1002/pds.1001

[13] B. Wu , M. Luo , F. Wu , Z. He , Y. Li , and T. Xu , “Acute Kidney Injury Associated With Remdesivir: A Comprehensive Pharmacovigilance Analysis of COVID‐19 Reports in FAERS,” Frontiers in Pharmacology 13 (2022): 692828. 35401177 10.3389/fphar.2022.692828 PMC8990823

[14] M. Gatti , I. C. Antonazzo , I. Diemberger , F. de Ponti , and E. Raschi , “Adverse Events With Sacubitril/Valsartan in the Real World: Emerging Signals to Target Preventive Strategies From the FDA Adverse Event Reporting System,” European Journal of Preventive Cardiology 28, no. 9 (2021): 983–989. 34402868 10.1177/2047487320915663

[15] S. Cecco , S. Puligheddu , M. Fusaroli , et al., “Emerging Toxicities of Antibody‐Drug Conjugates for Breast Cancer: Clinical Prioritization of Adverse Events From the FDA Adverse Event Reporting System,” Targeted Oncology 19, no. 3 (2024): 435–445. 38696126 10.1007/s11523-024-01058-9 PMC11111510

[16] S. Kinoshita , K. Hosomi , S. Yokoyama , and M. Takada , “Time‐To‐Onset Analysis of Amiodarone‐Associated Thyroid Dysfunction,” Journal of Clinical Pharmacy and Therapeutics 45, no. 1 (2020): 65–71. 31400296 10.1111/jcpt.13024

[17] F. Mazhar , V. Battini , M. Gringeri , et al., “The Impact of Anti‐TNFalpha Agents on Weight‐Related Changes: New Insights From a Real‐World Pharmacovigilance Study Using the FDA Adverse Event Reporting System (FAERS) Database,” Expert Opinion on Biological Therapy 21, no. 9 (2021): 1281–1290. 34191656 10.1080/14712598.2021.1948529

[18] L. La Mantia , L. M. Munari , and R. Lovati , “Glatiramer Acetate for Multiple Sclerosis,” Cochrane Database of Systematic Reviews 5 (2010): CD004678. 10.1002/14651858.CD004678.pub2 PMC13076001 20464733

[19] M. B. Bornstein , A. Miller , S. Slagle , et al., “A Pilot Trial of Cop 1 in Exacerbating‐Remitting Multiple Sclerosis,” New England Journal of Medicine 317, no. 7 (1987): 408–414. 3302705 10.1056/NEJM198708133170703

[20] B. Brochet , “Long‐Term Effects of Glatiramer Acetate in Multiple Sclerosis,” Revue Neurologique 164, no. 11 (2008): 917–926. 18790510 10.1016/j.neurol.2008.02.045

[21] M. B. Bornstein , A. Miller , S. Slagle , et al., “A Placebo‐Controlled, Double‐Blind, Randomized, Two‐Center, Pilot Trial of Cop 1 in Chronic Progressive Multiple Sclerosis,” Neurology 41, no. 4 (1991): 533–539. 2011253 10.1212/wnl.41.4.533

[22] R. J. Yu , M. S. Krantz , E. J. Phillips , et al., “Emerging Causes of Drug‐Induced Anaphylaxis: A Review of Anaphylaxis‐Associated Reports in the FDA Adverse Event Reporting System (FAERS),” Journal of Allergy and Clinical Immunology: In Practice 9, no. 2 (2021): 819–829. 32992044 10.1016/j.jaip.2020.09.021 PMC7870524

[23] M. Worm , S. Vieths , and V. Mahler , “An Update on Anaphylaxis and Urticaria,” Journal of Allergy and Clinical Immunology 150, no. 6 (2022): 1265–1278. 36481047 10.1016/j.jaci.2022.10.014

[24] M. Filippi , J. S. Wolinsky , and G. Comi , “Effects of Oral Glatiramer Acetate on Clinical and MRI‐Monitored Disease Activity in Patients With Relapsing Multiple Sclerosis: A Multicentre, Double‐Blind, Randomised, Placebo‐Controlled Study,” Lancet Neurology 5, no. 3 (2006): 213–220. 16488376 10.1016/S1474-4422(06)70327-1

[25] E. Amsler , J. E. Autegarden , H. Gaouar , C. Frances , and A. Soria , “Management of Immediate Hypersensitivity Reaction to Glatiramer Acetate,” European Journal of Dermatology 27, no. 1 (2017): 92–95. 27799134 10.1684/ejd.2016.2907

[26] D. Cadavid , J. Cheriyan , J. Skurnick , J. A. Lincoln , L. J. Wolansky , and S. D. Cook , “New Acute and Chronic Black Holes in Patients With Multiple Sclerosis Randomised to Interferon Beta‐1b or Glatiramer Acetate,” Journal of Neurology, Neurosurgery, and Psychiatry 80, no. 12 (2009): 1337–1343. 19687024 10.1136/jnnp.2008.171090

[27] B. C. Kieseier and O. Stuve , “A Critical Appraisal of Treatment Decisions in Multiple Sclerosis—Old Versus New,” Nature Reviews. Neurology 7, no. 5 (2011): 255–262. 21467994 10.1038/nrneurol.2011.41

[28] J. A. Cohen , F. Barkhof , G. Comi , et al., “Oral Fingolimod or Intramuscular Interferon for Relapsing Multiple Sclerosis,” New England Journal of Medicine 362, no. 5 (2010): 402–415. 20089954 10.1056/NEJMoa0907839

