# Supplementary material for: Reported Characteristics of Anaphylaxis Associated With Glatiramer Acetate: A Pharmacovigilance Analysis of the FDA and Canadian Databases
Source: Pharmacol Res Perspect. 2026 Jul 31;14(4):e70303. doi: 10.1002/prp2.70303 (PMC13428025; doi:10.1002/prp2.70303)
Supplement: Supplementary file 1 — Table S1: Calculation of reporting odds ratio (ROR). Table S2: Criteria and relevant scores to prioritize AEs emerged from disproportionality analysis. Table S3: Anaphylactic PTs from MedDRA, version 22.1. Table S4: Signal strength of reports of glatiramer at the System Organ Class (SOC) level in the FAERS database. Table S5: Clinical priority assessment of results for disproportionality signals in the CAVR database. Table S6: Signal strength of reports of glatiramer at the System Organ Class (SOC) level in the CAVR database. [file PRP2-14-e70303-s001.docx]

Supplementary Material

**Supplementary Table 1**. Calculation of reporting odds ratio (ROR).

|  | Reports with target AE | Reports without target AE |
| --- | --- | --- |
| Reports with semaglutide | a | b |
| Reports without semaglutide | c | d |

a, number of reports containing both the target drug (glatiramer) and target AE; b, number of reports containing other AEs of the target drug; c, number of reports containing the target AE of other drugs; d, number of reports containing other drugs and other AEs.

AEs, Adverse Events; ROR, Reporting Odds Ratio; CI, confidence interval.

The calculation formulas are shown below:

1. ROR=ad/b/c
2. 95%CI=e^ln(ROR)±1.96(1/a+1/b+1/c+1/d)^0.5^

**Supplementary Table 2**. Criteria and relevant scores to prioritise AEs emerged from disproportionality analysis.

| Criterium | 2 points | 1 point | 0 point |
| --- | --- | --- | --- |
| Reporting rate (cases/non-cases) | > 10% | 1 – 10% | 0 – 1% |
| Signal stability (consistency across disproportionality analyses) | 3 of 3 | 2 of 3 | 1 of 3 |
| Reported case fatality rate (proportion of reports with death as outcome) | > 50% | 25–50% | < 25% |
| Clinical relevance (serious likely drug-attributable AEs) | DME | IME | None |

AEs, adverse events; DME, designated medical event; IME, important medical event. A score of 0-2, 3-5, and 6-8 was identified, respectively, for AEs with weak, moderate, or strong priority.

**Supplementary Table 3.** Anaphylactic PTs from MedDRA, version 22.1

| PT | Code | SOC |
| --- | --- | --- |
| Acquired C1 inhibitor deficiency | 10081035 | Skin and subcutaneous tissue disorders |
| Acute respiratory failure | 10001053 | Respiratory, thoracic and mediastinal disorders |
| Allergic oedema | 10060934 | Immune system disorders |
| Anaphylactic reaction | 10002198 | Immune system disorders |
| Anaphylactic shock | 10002199 | Immune system disorders |
| Anaphylactic transfusion reaction | 10067113 | Injury, poisoning and procedural complications |
| Anaphylactoid reaction | 10002216 | Immune system disorders |
| Anaphylactoid shock | 10063119 | Immune system disorders |
| Angioedema | 10002424 | Skin and subcutaneous tissue disorders |
| Angioedema | 10002424 | Skin and subcutaneous tissue disorders |
| Asthma | 10003553 | Respiratory, thoracic and mediastinal disorders |
| Blood pressure decreased | 10005734 | Investigations |
| Blood pressure diastolic decreased | 10005737 | Investigations |
| Blood pressure systolic decreased | 10005758 | Investigations |
| Bronchial oedema | 10056695 | Respiratory, thoracic and mediastinal disorders |
| Bronchospasm | 10006482 | Respiratory, thoracic and mediastinal disorders |
| Cardiac arrest | 10007515 | Cardiac disorders |
| Cardio-respiratory arrest | 10007617 | Cardiac disorders |
| Cardio-respiratory distress | 10049874 | Cardiac disorders |
| Cardiovascular insufficiency | 10065929 | Cardiac disorders |
| Chest discomfort | 10008469 | General disorders and administration site conditions |
| Choking | 10008589 | Respiratory, thoracic and mediastinal disorders |
| Choking sensation | 10008590 | Respiratory, thoracic and mediastinal disorders |
| Circulatory collapse | 10009192 | Vascular disorders |
| Circumoral oedema | 10052250 | Skin and subcutaneous tissue disorders |
| Circumoral swelling | 10081703 | Skin and subcutaneous tissue disorders |
| Cough | 10011224 | Respiratory, thoracic and mediastinal disorders |
| Cough variant asthma | 10063076 | Respiratory, thoracic and mediastinal disorders |
| Cyanosis | 10011703 | Vascular disorders |
| Dialysis membrane reaction | 10076665 | Immune system disorders |
| Dialysis membrane reaction | 10076665 | Immune system disorders |
| Diastolic hypotension | 10066077 | Vascular disorders |
| Dyspnoea | 10013968 | Respiratory, thoracic and mediastinal disorders |
| Dyspnoea | 10013968 | Respiratory, thoracic and mediastinal disorders |
| Erythema | 10015150 | Skin and subcutaneous tissue disorders |
| Erythema | 10015150 | Skin and subcutaneous tissue disorders |
| Eye oedema | 10052139 | Eye disorders |
| Eye pruritus | 10052140 | Eye disorders |
| Eye swelling | 10015967 | Eye disorders |
| Eyelid oedema | 10015993 | Eye disorders |
| Face oedema | 10016029 | General disorders and administration site conditions |
| Fixed eruption | 10016741 | Skin and subcutaneous tissue disorders |
| Fixed eruption | 10016741 | Skin and subcutaneous tissue disorders |
| Flushing | 10016825 | Vascular disorders |
| Hereditary angioedema with C1 esterase inhibitor deficiency | 10080955 | Congenital, familial and genetic disorders |
| Hyperventilation | 10020910 | Respiratory, thoracic and mediastinal disorders |
| Hypotension | 10021097 | Vascular disorders |
| Hypotensive crisis | 10083659 | Vascular disorders |
| Injection site urticaria | 10022107 | General disorders and administration site conditions |
| Irregular breathing | 10076213 | Respiratory, thoracic and mediastinal disorders |
| Kounis syndrome | 10069167 | Cardiac disorders |
| Laryngeal dyspnoea | 10052390 | Respiratory, thoracic and mediastinal disorders |
| Laryngeal oedema | 10023845 | Respiratory, thoracic and mediastinal disorders |
| Laryngospasm | 10023891 | Respiratory, thoracic and mediastinal disorders |
| Laryngotracheal oedema | 10023893 | Respiratory, thoracic and mediastinal disorders |
| Lip oedema | 10024558 | Gastrointestinal disorders |
| Lip swelling | 10024570 | Gastrointestinal disorders |
| Mouth swelling | 10075203 | Gastrointestinal disorders |
| Nasal obstruction | 10028748 | Respiratory, thoracic and mediastinal disorders |
| Nodular rash | 10075807 | Skin and subcutaneous tissue disorders |
| Ocular hyperaemia | 10030041 | Eye disorders |
| Ocular hyperaemia | 10030041 | Eye disorders |
| Oedema | 10030095 | General disorders and administration site conditions |
| Oedema | 10030095 | General disorders and administration site conditions |
| Oedema blister | 10080039 | Skin and subcutaneous tissue disorders |
| Oedema mouth | 10030110 | Gastrointestinal disorders |
| Oropharyngeal oedema | 10078783 | Respiratory, thoracic and mediastinal disorders |
| Oropharyngeal spasm | 10031111 | Respiratory, thoracic and mediastinal disorders |
| Oropharyngeal swelling | 10031118 | Respiratory, thoracic and mediastinal disorders |
| Periorbital oedema | 10034545 | Eye disorders |
| Periorbital swelling | 10056647 | Eye disorders |
| Pharyngeal oedema | 10034829 | Respiratory, thoracic and mediastinal disorders |
| Pharyngeal swelling | 10082270 | Respiratory, thoracic and mediastinal disorders |
| Post procedural hypotension | 10084013 | Injury, poisoning and procedural complications |
| Post procedural hypotension | 10084013 | Injury, poisoning and procedural complications |
| Procedural shock | 10080894 | Injury, poisoning and procedural complications |
| Pruritus | 10037087 | Skin and subcutaneous tissue disorders |
| Pruritus allergic | 10063438 | Skin and subcutaneous tissue disorders |
| Rash | 10037844 | Skin and subcutaneous tissue disorders |
| Rash erythematous | 10037855 | Skin and subcutaneous tissue disorders |
| Rash pruritic | 10037884 | Skin and subcutaneous tissue disorders |
| Respiratory arrest | 10038669 | Respiratory, thoracic and mediastinal disorders |
| Respiratory distress | 10038687 | Respiratory, thoracic and mediastinal disorders |
| Respiratory dyskinesia | 10057570 | Nervous system disorders |
| Respiratory failure | 10038695 | Respiratory, thoracic and mediastinal disorders |
| Reversible airways obstruction | 10062109 | Respiratory, thoracic and mediastinal disorders |
| Sensation of foreign body | 10061549 | General disorders and administration site conditions |
| Shock | 10040560 | Vascular disorders |
| Shock symptom | 10040581 | Vascular disorders |
| Skin swelling | 10053262 | Skin and subcutaneous tissue disorders |
| Sneezing | 10041232 | Respiratory, thoracic and mediastinal disorders |
| Stridor | 10042241 | Respiratory, thoracic and mediastinal disorders |
| Swelling | 10042674 | General disorders and administration site conditions |
| Swelling face | 10042682 | General disorders and administration site conditions |
| Swelling of eyelid | 10042690 | Eye disorders |
| Swollen tongue | 10042727 | Gastrointestinal disorders |
| Tachypnoea | 10043089 | Respiratory, thoracic and mediastinal disorders |
| Throat tightness | 10043528 | Respiratory, thoracic and mediastinal disorders |
| Tongue oedema | 10043967 | Gastrointestinal disorders |
| Tracheal obstruction | 10044291 | Injury, poisoning and procedural complications |
| Tracheal oedema | 10044296 | Respiratory, thoracic and mediastinal disorders |
| Type I hypersensitivity | 10045240 | Immune system disorders |
| Upper airway obstruction | 10067775 | Respiratory, thoracic and mediastinal disorders |
| Urticaria | 10046735 | Skin and subcutaneous tissue disorders |
| Urticaria papular | 10046750 | Skin and subcutaneous tissue disorders |
| Vaccine associated enhanced respiratory disease | 10085001 | NA |
| Wheezing | 10047924 | Respiratory, thoracic and mediastinal disorders |

**Supplementary Table 4**. Signal strength of reports of glatiramer at the System Organ Class (SOC) level in the FAERS database.

| **SOC/Target PTs** | n | ROR (95%Cl) |
| --- | --- | --- |
| Non-Anaphylatic Reaction | 123771 | 0.53 (0.52 - 0.54) |
| General Disorders and Administration Site Conditions* | 48708 | 2.58 (2.55 - 2.61) |
| Nervous System Disorders* | 22221 | 2.07 (2.04 - 2.1) |
| **Anaphylatic Reaction*** | 15755 | 1.88 (1.85 - 1.91) |
| Injury, Poisoning and Procedural Complications | 9350 | 0.62 (0.61 - 0.64) |
| Skin and Subcutaneous Tissue Disorders* | 8170 | 1.1 (1.08 - 1.13) |
| Gastrointestinal Disorders | 5991 | 0.49 (0.47 - 0.5) |
| Musculoskeletal and Connective Tissue Disorders | 5893 | 0.81 (0.79 - 0.83) |
| Respiratory, Thoracic and Mediastinal Disorders | 5238 | 0.8 (0.78 - 0.82) |
| Infections and Infestations | 4200 | 0.57 (0.55 - 0.58) |
| Psychiatric Disorders | 4150 | 0.52 (0.5 - 0.54) |
| Investigations | 3402 | 0.39 (0.37 - 0.4) |
| Immune System Disorders* | 2913 | 1.93 (1.86 - 2) |
| Vascular Disorders | 2822 | 0.96 (0.92 - 0.99) |
| Eye Disorders | 2180 | 0.79 (0.76 - 0.82) |
| Cardiac Disorders | 2055 | 0.56 (0.53 - 0.58) |
| Product Issues | 2041 | 0.91 (0.87 - 0.95) |
| Surgical and Medical Procedures | 1909 | 1.02 (0.98 - 1.07) |
| Neoplasms Benign, Malignant and Unspecified | 1493 | 0.4 (0.38 - 0.43) |
| Renal and Urinary Disorders | 1008 | 0.38 (0.35 - 0.4) |
| Pregnancy, Puerperium and Perinatal Conditions* | 909 | 1.54 (1.44 - 1.64) |
| Social Circumstances | 687 | 1.07 (0.99 - 1.15) |
| Metabolism and Nutrition Disorders | 647 | 0.21 (0.2 - 0.23) |
| Ear and Labyrinth Disorders | 565 | 0.94 (0.87 - 1.03) |
| Blood and Lymphatic System Disorders | 481 | 0.2 (0.18 - 0.22) |
| Hepatobiliary Disorders | 463 | 0.36 (0.33 - 0.4) |
| Reproductive System and Breast Disorders | 440 | 0.36 (0.32 - 0.39) |
| Congenital, Familial and Genetic Disorders | 229 | 0.53 (0.47 - 0.6) |
| Endocrine Disorders | 133 | 0.38 (0.32 - 0.45) |

* indicates statistically significant signals in the algorithm. ROR, reporting odds ratio; CI, confidence interval.

**Supplementary Table 5**. Clinical priority assessment of results for disproportionality signals in the CAVR database.

| **PT (Preferred Term)** | **Glatiraner** | **ROR** | **Death (n)** | **Priority level (score)** |
| --- | --- | --- | --- | --- |
| Injection Site Urticaria | 12 | 22.11 | 0 | Weak (2) |
| Throat Tightness | 53 | 16.14 | 1 | Moderate (3) |
| Pharyngeal Swelling | 21 | 9.37 | 0 | Weak (2) |
| Swelling Face | 41 | 8.94 | 0 | Weak (2) |
| Lip Swelling | 26 | 7.61 | 0 | Weak (2) |
| Flushing | 68 | 7.05 | 0 | Moderate (3) |
| Face Oedema | 19 | 6.14 | 0 | Weak (2) |
| Chest Discomfort | 70 | 5.83 | 0 | Moderate (3) |
| Eye Swelling | 12 | 4.71 | 0 | Weak (2) |
| Cyanosis | 7 | 4.53 | 0 | Weak (2) |
| Respiratory Arrest | 4 | 4.49 | 0 | Moderate (3) |
| Swollen Tongue | 12 | 4.45 | 0 | Weak (2) |
| Choking | 6 | 4.01 | 1 | Moderate (3) |
| Erythema | 75 | 3.59 | 0 | Moderate (3) |
| Dyspnoea | 172 | 3.51 | 0 | Moderate (3) |
| Anaphylactic Reaction | 20 | 3.39 | 0 | Moderate (4) |
| Anaphylactic Shock | 4 | 2.81 | 0 | Moderate (3) |
| Urticaria | 64 | 2.02 | 0 | Weak (2) |

**Supplementary Table 6**. Signal strength of reports of glatiramer at the System Organ Class (SOC) level in the CAVR database.

| **SOC/Target PTs** | n | ROR (95%Cl) |
| --- | --- | --- |
| Non-Anaphylactic Reaction | 3588 | 0.4 (0.37 - 0.43) |
| Pregnancy, Puerperium and Perinatal Conditions* | 43 | 3.5 (2.59 - 4.73) |
| Immune System Disorders* | 117 | 2.61 (2.17 - 3.13) |
| **Anaphylactic Reaction*** | 686 | 2.52 (2.34 - 2.72) |
| Nervous System Disorders* | 644 | 2.22 (2.04 - 2.41) |
| Vascular Disorders* | 159 | 1.7 (1.45 - 1.99) |
| Congenital, Familial and Genetic Disorders* | 9 | 1.68 (0.87 - 3.23) |
| General Disorders and Administration Site Conditions* | 1224 | 1.47 (1.38 - 1.57) |
| Cardiac Disorders* | 115 | 1.45 (1.21 - 1.75) |
| Respiratory, Thoracic and Mediastinal Disorders* | 334 | 1.31 (1.17 - 1.46) |
| Skin And Subcutaneous Tissue Disorders* | 301 | 1.21 (1.08 - 1.36) |
| Neoplasms Benign, Malignant and Unspecified* | 68 | 1 (0.78 - 1.27) |
| Psychiatric Disorders | 177 | 0.99 (0.85 - 1.15) |
| Eye Disorders | 69 | 0.97 (0.76 - 1.23) |
| Endocrine Disorders | 7 | 0.88 (0.42 - 1.84) |
| Ear And Labyrinth Disorders | 14 | 0.76 (0.45 - 1.29) |
| Renal And Urinary Disorders | 38 | 0.74 (0.54 - 1.02) |
| Hepatobiliary Disorders | 20 | 0.73 (0.47 - 1.13) |
| Reproductive System And Breast Disorders | 18 | 0.66 (0.41 - 1.05) |
| Gastrointestinal Disorders | 302 | 0.65 (0.58 - 0.73) |
| Social Circumstances | 12 | 0.64 (0.37 - 1.14) |
| Injury, Poisoning and Procedural Complications | 208 | 0.55 (0.48 - 0.63) |
| Musculoskeletal And Connective Tissue Disorders | 168 | 0.54 (0.47 - 0.63) |
| Infections And Infestations | 173 | 0.52 (0.45 - 0.61) |
| Blood And Lymphatic System Disorders | 25 | 0.46 (0.31 - 0.68) |
| Product Issues | 17 | 0.44 (0.27 - 0.71) |
| Surgical And Medical Procedures | 23 | 0.4 (0.27 - 0.61) |
| Investigations | 133 | 0.37 (0.31 - 0.43) |
| Metabolism And Nutrition Disorders | 19 | 0.27 (0.17 - 0.42) |

* indicates statistically significant signals in the algorithm. ROR, reporting odds ratio; CI, confidence interval.
